# Harmonizing leadership: Blending transactional and transformational styles in pharmacy practice

**DOI:** 10.1177/17151635241253593

**Published:** 2024-05-20

**Authors:** Liam Jackman

**Affiliations:** Institute of Health Policy, Management and Evaluation and the Temerty Faculty of Medicine, University of Toronto, Toronto, Ontario

Transactional and transformational leadership styles are often thought of as opposites of one another. There is a prevailing misconception that one must choose between these styles when, in fact, successful leadership finds itself in the integration of the two.^1^ In the dynamic landscape of pharmacy, this duality is essential, as transactional approaches allow leaders to maintain stability within their teams, ensuring efficient day-to-day operations. Meanwhile, transformational approaches allow leaders to embrace change, such as advancements to scope of practice, and help their team members seamlessly integrate these changes into their practices.

In this article, I will examine the knowledge and skills that one needs to acquire to effectively use these styles in tandem and I will outline strategies that one can use to do so.

The transactional leadership model is based on exchange dynamics, whereby the leader employs strategies such as rewards and penalties to influence team members’ behaviours. The transactional leader rewards those who perform to a set standard and penalizes those who fail to perform to this standard. The actual approach adheres to 1 of the following subdimensions: contingent reward, active management by exception or passive management by exception. In contingent reward, the leader communicates performance expectations and the criteria used to determine the provision of rewards.^2^ In active management by exception, the leader monitors performance and intervenes when problems arise.^3^ In passive management by exception, the leader monitors performance but intervenes only when the problems that arise become significant.^3^ Ultimately, this leader operates within the existing organizational culture, promoting compliance with existing organizational goals.^4^

In a health care setting, such as pharmacy, transactional leadership finds its place in promoting adherence to measures such as hand hygiene, use of personal protective equipment, infection control, safeguarding of medications and accurate documentation, which are important across all patient demographics. This approach can also be used to encourage the efficient use of health care resources, including diagnostic procedures and therapeutic interventions. The use of a transactional leadership approach in these contexts ensures that patients receive care in line with established policies and procedures. Ultimately, this ensures that care is safe for patients and sustainable for organizations.

To effectively implement transactional leadership and drive improvements for patients, health care providers and health care settings, there is a need to develop specific skills, including but not limited to communication skills, organizational skills, performance management skills and incentive design skills. Transactional leaders set clear roles and responsibilities for their team members and need strong communication skills to convey this information. They set up systems that facilitate the execution of assigned responsibilities and as such need strong organizational skills. They assess performance against predefined standards, acknowledge achievement and address nonachievement and as such need strong performance management skills. They also administer incentives and need to select those that resonate with their teams, therefore requiring strong incentive design skills.

To cultivate communication skills, one can participate in workshops that provide opportunities for role-play–relevant conversations. These role-play scenarios serve as opportunities to prepare and practise delivering^5^ important information while receiving feedback and refining the approach accordingly. To cultivate organizational skills, one can participate in workshops that teach the use of project management software to help delegate, set deadlines and monitor progress. One can also participate in workshops on process mapping to help better understand workflows, specifically where issues arise, and what changes could be made to enhance overall efficiency.^6^ To cultivate performance management skills, one can learn to conduct comprehensive performance reviews, which encompass a balanced assessment of both strengths and weaknesses. The incorporation of coaching techniques can enhance the effectiveness of these performance reviews by facilitating the implementation of recommended changes into practical actions. Finally, to cultivate incentive design skills, one can learn about motivational theories to better understand what incentivizes others and use these insights to implement relevant incentives within their teams.

The transformational leadership model holds the power to transcend basic exchange dynamics. It is important to lead in a manner that nurtures a culture of compassion and collective commitment to patient care. As Kotter^7^ has famously pointed out, “organizations are over-managed and under-led.” Transformational leadership ventures into the domains of inspiration and visionary thinking, encapsulating subdimensions of idealized influence, inspirational motivation, intellectual stimulation and individualized consideration.^8^

The primary subdimension of transformational leadership, idealized influence, involves eliciting followers’ admiration and respect, motivating them to emulate the leader.^9^ The leader does this by modelling the behaviours that they expect from their team members and supporting their success, caring as much for them as they do for themselves. To cultivate idealized influence, one can learn about emotional intelligence by, for instance, participating in emotional intelligence workshops. One can also participate in team-building exercises to enhance trust-building skills.^10^

The second subdimension, inspirational motivation, involves cultivating a commitment to a shared vision.^9^ The leader does this by coupling individuals’ contributions with the organization’s success, thereby giving meaning to their work.^11^ To cultivate inspirational motivation, one can learn about motivational theories to tap into team members’ intrinsic motivations and inspire their commitment.

The third subdimension, intellectual stimulation, spurs innovation by encouraging critical thinking and the creation of creative solutions.^9^ The leader does this by demonstrating courage, specifically the courage to try something new and the courage to trust others, both of which carry risks.^12^ To cultivate intellectual stimulation, one can be open to opportunities to try something new and, in turn, increase risk threshold. One can also collaborate with others to better contextualize risks, both the risks of challenging the status quo and the risks of maintaining it.

The fourth subdimension, individualized consideration, involves providing person-specific support.^9^ One can learn about coaching to better cater to diverse learning styles. One can also learn about motivational interviewing to create person-specific professional development plans based on unique interests. This approach considers individual needs and maximizes potential.

We should all aspire to be adaptive leaders who integrate transactional and transformational leadership styles (Table 1). It is through embracing the strengths of both that pharmacy leaders can create a more adaptable, responsive and ultimately more effective practice environment. This synergy empowers leaders to maintain the highest standards of patient care while fostering an atmosphere of continuous improvement and professional development. In the ever-evolving field of pharmacy, the fusion of transactional and transformational leadership moves beyond theoretical ideals, becoming an essential practice for success.

**Table 1:** Transactional vs transformational leadership attributes^13,14^

Attribute	Transactional leadership	Transformational leadership
Primary goal	Pursue efficiency and maintain stability	Foster innovation and encourage growth
Motivation	Driven by external rewards and penalties	Driven by internal sense of purpose
Communication	Directive	Dialogue encouraged
Leader’s role	Acts as a manager enforcing rules	Acts as a mentor providing guidance
Decision-making	Makes decisions alone	Makes decisions collaboratively
Employee development	Focuses on training for specific skills	Focuses on long-term personal and professional development
Approach to change	Maintains the status quo	Initiates and embraces change
Expected outcome	Predictability and order	Creativity and organizational transformation
